# Using Nanopolymeric Microspheres for Enhanced Oil Recovery in a High-Salt Oil Reservoir in B‑3 Block, Erdos Basin, China

**DOI:** 10.1021/acsomega.6c00424

**Published:** 2026-03-24

**Authors:** Xinyi Sun, Hongda Hao, Yu Jiang, Song Deng, Mingguo Peng, Qiu Li, Chengguo Liu, Xiaopeng Yan, Ming Qu

**Affiliations:** † School of Petroleum and Natural Gas Engineering, School of Energy, 12412 Changzhou University, Changzhou 213164, China; ‡ Geological Engineering Research Institute, Changqing Industrial Group Changqing Oilfield Company, Changqing 710018, China; § Sanya Offshore Oil & Gas Research Institute, Northeast Petroleum University, Sanya 572024, China

## Abstract

To address the challenges of inefficient water flooding and low oil recovery in high-salinity, low-permeability reservoirs of the B-3 Block, Erdos Basin, this study developed a novel temperature-resistant and salt-tolerant nanosphere system based on an acrylamide (AM)/2-acrylamido-2-methylpropanesulfonic acid (AMPS) copolymer. Systematic physical simulation experiments were conducted to evaluate the performance and enhanced oil recovery (EOR) potential of these nanospheres under high-temperature and high-salinity conditions. Results demonstrate that the nanosphere system retains excellent swelling stability and plugging capacity at a salinity of 23,800 mg/L and 50 °C while achieving effective injectivity and in situ migration within cores possessing pore-throat sizes of 10–300 × 10^–3^ μm^2^. Oil displacement experiments confirmed that, compared to conventional water flooding (with a recovery factor of ∼45%), the nanosphere-assisted process significantly reduced water cut by 5–10% and increased the ultimate oil recovery by 15–17%. The underlying EOR mechanism is primarily attributed to effective blockage of high-permeability channels and diversion of subsequent injection fluid, thereby improving sweep efficiency. The study also identified the optimal injection parameters: a concentration of 1250 mg/L, a rate of 0.3 mL/min, and a volume of 1.0 PV, which, under conditions of a permeability of 60.61 × 10^–3^ μm^2^ and a permeability contrast of 2.29, yielded the most pronounced profile control and displacement effect. This work demonstrates that the developed nanosphere system offers a robust and effective technical solution for the efficient development of challenging high-salinity, low-permeability reservoirs.

## Graphical abstract

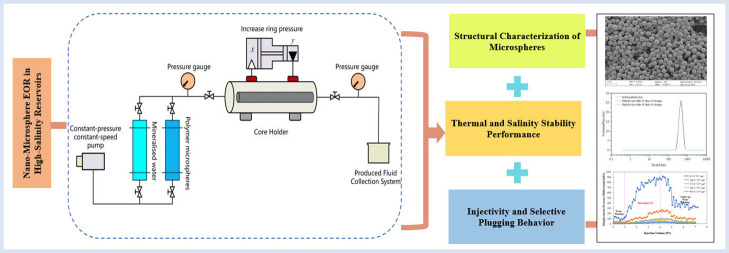

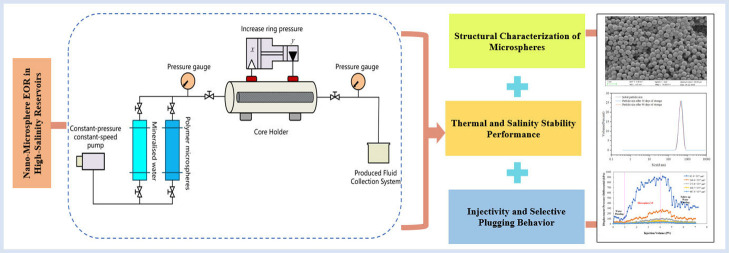

## 1 Introduction

As conventional reservoirs enter high-water-content phases, efficient development of high-salinity, low-permeability reservoirs has become a critical challenge for enhancing oil recovery.^1^ Such reservoirs typically exhibit high mineralization (tens of thousands to hundreds of thousands of mg/L), narrow pores and throats, and strong heterogeneity, leading to degradation or failure of conventional chemical flooding agents in the reservoir.^2^ Simultaneously, injected water often flows along high-permeability pathways, resulting in a low sweep efficiency. For such reservoirs, modified polymer flooding, surfactant flooding, composite flooding, and deep profile-control techniques have been extensively studied. Among these, polymer flooding is the most common due to its extensive laboratory and field researches.^3–8^

However, conventional polymers face two major challenges in high-salinity, low-permeability reservoirs: first, high solution viscosity and poor microfracture throat passage lead to high-injection pressure and ineffective injection; second, chemical degradation readily occurs under high-temperature, high-salinity conditions. Together, these factors severely limit their oil displacement efficiency.^9–11^ Conventional polymers such as HPAM mainly enhance oil recovery by increasing the viscosity of the injected water, thereby improving the mobility ratio and microscopic displacement efficiency. Their effectiveness depends on maintaining sufficient molecular chain extension and solution viscosity under the reservoir conditions. In contrast, polymer microspheres function through a particle-based profile-control mechanism rather than bulk viscosity modification.^12^ Due to their cross-linked structure, microspheres can swell after injection and selectively accumulate in high-permeability channels through deformation and bridging, diverting subsequent fluids into unswept zones.^13,14^ Therefore, conventional polymers primarily improve microscopic displacement efficiency via mobility control, whereas polymer microspheres mainly enhance macroscopic sweep efficiency through selective plugging and flow diversion, making them more suitable for heterogeneous and high-salinity reservoirs.^15,16^

In certain oilfields, reservoirs are deeply buried with high pressure, high temperatures, and high-salinity water. Furthermore, the utilization of produced water reinjection also poses challenges for conventional polymer microspheres, whose hydration and swelling capabilities are limited under high-temperature and high-salinity conditions.^17–19^ These microspheres struggle to form stable structures, are prone to dehydration and degradation, and exhibit poor long-term stability, failing to meet the demands of deep profile control. On one hand, high mineralization induces an “antipolyelectrolyte effect”, inhibiting microsphere swelling and reducing plugging strength; multivalent ions disrupt microsphere network structures, compromising long-term stability; and conventional microspheres struggle to migrate through low-permeability pore throats, often causing near-wellbore blockages. On the other hand, microspheres tend to swell prematurely under high temperatures, hindering effective deep profile control.^20,21^ Consequently, researchers have proposed various modification strategies, including introducing sulfonic acid monomers (e.g., AMPS), optimizing cross-linking systems, and developing novel emulsifiers. Studies indicate that incorporating AMPS monomers significantly enhances the salt resistance of polymer microspheres.^22^ Bai et al.^23^ successfully prepared polymer microspheres with high viscosity and excellent dispersion stability by copolymerizing acrylamide (AM) with 2-acrylamido-2-methylpropanesulfonic acid (AMPS), achieving an average particle size of approximately 1 μm. Researchers further explored the incorporation of other functional monomers to endow microspheres with properties tailored to specific oilfield requirements. Zhang et al.^24^ developed novel temperature-resistant, delayed-swelling polymer microspheres by introducing cationic monomer DAC and thermally stable monomer NVP, aiming to enhance oil recovery in low-permeability reservoirs. Cross-linking agents also play a crucial role in microsphere preparation and performance regulation.^25,26^ Lei et al.^27^ introduced NAM as a thermally initiated self-cross-linking agent and AMPS as a reactive emulsifier. Using a semicontinuous soap-free emulsion polymerization process with MMA and St as raw materials, they synthesized a series of self-cross-linking emulsions and applied them for the first time in water-based drilling fluids. Traditional polymer microspheres are predominantly prepared via single-step polymerization. Zhang et al.^28^ employed a two-step polymerization approach to successfully synthesize thermosensitive, core–shell structured superabsorbent microspheres. The resulting samples exhibited excellent thermal stability and water absorption properties at elevated temperatures (80 °C). However, high shear forces during injection can disrupt polymer chains, reducing solution viscosity and impairing oil displacement efficiency.^29^ To address this, researchers explored reverse emulsion polymerization for preparing oil displacement agents that dissolve and inject directly into reservoirs without prior solidification. Additionally, the oilfield site generates substantial produced water, causing significant environmental pollution. Effectively using this wastewater to prepare polymer microspheres under wastewater conditions holds great significance for environmental protection and resource recycling. However, preparing polymer microspheres under wastewater conditions presents challenges such as insufficient system viscosity and inability to maintain ultralow interfacial tension, making the process difficult.^30,31^

This study focuses on the B-3 Block of the Maling Oilfield in the Ordos Basin. Since its production commencement in 1998, the primary producing interval has been the Yan 1012 Member of the Yan’an Formation. Although the block contains recoverable geological reserves of 2.5912 × 10^6^ t, the waterflood recovery factor remains as low as 27.7%. The reservoir is characterized by an average permeability of 63.2 × 10^–3^ μm^2^, a high formation water salinity of 23,800 mg/L, Ca^2+^ and Mg^2+^ concentrations of 581 mg/L, and pronounced heterogeneity. Under such conditions, conventional water flooding and traditional profile-control treatments fail to effectively expand sweep volume or achieve stable flow diversion, necessitating the development of advanced functional materials with enhanced temperature resistance, salt tolerance, and deep profile-control capability. In response to these engineering challenges, this study innovatively establishes a multiscale integrated framework linking molecular structural design, pore-scale interaction mechanisms, and macroscopic displacement performance. Within this framework, a temperature-resistant and salt-tolerant AM/AMPS-based nanopolymeric microsphere system was developed via reverse emulsion polymerization. By synergistically coupling the thermally stable acrylamide (AM) backbone with the sulfonic acid functionality of 2-acrylamido-2-methylpropanesulfonic acid (AMPS), long-term structural stability and functional retention were achieved under high-salinity conditions of 23,800 mg/L.

The innovation of this work is reflected in three key aspects. At the material-design level, a synergistic monomer engineering strategy enables the integrated regulation of thermal resistance and salt tolerance within a unified molecular architecture, overcoming the conventional single-performance optimization paradigm. At the mechanistic level, the study systematically elucidates how pore-scale bridging and elastic particle deformation behaviors are transmitted across scales and converted into macroscopic flow diversion and sweep-volume expansion over a wide range of permeability, thereby establishing an intrinsic linkage between microscopic interactions and macroscopic profile-control performance. At the engineering-evaluation level, an integrated technical system incorporating structural characterization, injectivity assessment, migration analysis, selective plugging evaluation, and operational parameter optimization was constructed, quantitatively bridging polymer molecular design and reservoir-scale displacement efficiency. By integrating polymer chemistry principles, porous media flow theory, and reservoir engineering optimization, this study provides a transferable technical pathway and a solid theoretical foundation for the efficient development of high-salinity, low-permeability reservoirs.

## 2 Materials and Experiments

### 2.1 Preparation and Characterization of Microspheres

#### 2.1.1 Preparation Method of Microspheres

Acrylamide, 2-acrylamido-2-methylpropanesulfonic acid, ethylenediaminetetraacetate, and *N*,*N′*-methylenebis­(acrylamide) are used for the preparation of antisalt Microspheres, and the specific experimental procedure is as follows:

(1) Dissolve acrylamide (AM) and 2-acrylamido-2-methylpropanesulfonic acid (AMPS) in deionized water at a molar ratio of 7:3. Add a small amount of disodium ethylenediaminetetraacetate (EDTA) to chelate metal ions and the cross-linking agent *N*,*N*′-methylenebis­(acrylamide) (MBA). Stir until completely dissolved and form the aqueous phase. Add NaOH to adjust the pH to 7 and incorporate a portion of the initiator potassium persulfate (K_2_S_2_O_8_).
(2) Dissolve appropriate amounts of emulsifiers, sorbitan monolaurate (Span-80) and polysorbate-80 (Tween-80), at a mass ratio of 8:5 in No. 3 white oil. Add oil-soluble initiator azobis­(isobutyronitrile) (AIBN) and mix thoroughly with magnetic stirring.
(3) Slowly add the prepared aqueous phase to the oil phase. Transfer the mixture to a four-neck flask equipped with a mechanical stirrer, a thermometer, a condenser, and a nitrogen inlet. Initiate stirring at the preset speed (rpm) to form a water-in-oil (W/O) emulsion. Pass high-purity nitrogen gas for approximately 20–30 min to remove dissolved oxygen.
(4) Under nitrogen protection and continuous stirring, slowly add the remaining initiator, potassium persulfate solution, and reducing agent sodium sulfite (Na_2_SO_3_) aqueous solution (forming the redox initiation system) using a constant-pressure dropping funnel. After completion, slowly raise the temperature to the preset reaction temperature. Maintain the reaction at this temperature for a specified duration, observing changes in system viscosity and emulsion state;
(5) After reaction completion, cool the system to room temperature and add excess anhydrous ethanol to break the emulsion, causing microspheres to precipitate. Finally, perform post-treatment: filter the precipitate under vacuum and wash it several times with petroleum ether and anhydrous ethanol alternately to remove residues. Place the obtained wet microspheres in a vacuum drying oven and dry them at 40–50 °C until constant weight is reached. The resulting polymer microsphere powder is stored in a desiccator for future use.

#### 2.1.2 Characterization Methods for Microspheres

#### 2.1.3 Morphological and Chemical Structure Characterization of Polymer Microspheres

Surface morphology and overall structural features of the microspheres were observed by using a German ZEISS Sigma360 scanning electron microscope (SEM). Before testing, the dried microsphere powder underwent gold spraying to enhance the conductivity. Images were captured at magnifications of 100 nm, 200 nm, 300 nm, 500 nm, 1 μm, 10 μm, 50 μm, 100 μm, and 500 μm. SEM imaging was used to evaluate the microsphere sphericity, surface smoothness, structural integrity, and presence of defects or agglomerates. Chemical structure characterization was performed by using a Thermo Fisher Scientific Nicolet iS20 Fourier Transform Infrared Spectrometer (FTIR). A small amount of microsphere powder (1–2 mg) was mixed with potassium bromide powder (100–200 mg), ground into a fine powder, and evenly distributed within a mold. The mixture was compressed into a transparent thin film, placed on the FTIR holder, and scanned over the range of 400–4000 cm^–1^ to identify characteristic functional groups and chemical structures within the microspheres.

#### 2.1.4 Thermal Stability Evaluation

The thermal decomposition behavior of microspheres was evaluated by using a PerkinElmer STA 8000 thermogravimetric analyzer (TG) under a nitrogen atmosphere. The heating rate was set at 10 °C/min, and the mass loss curves of the microsphere powder samples were recorded during the heating process from 30 to 800 °C. Analysis of the TG curves enabled determination of the microspheres’ initial decomposition temperature (*T*_5_%, corresponding to 5% weight loss), primary decomposition temperature range, maximum decomposition rate temperature (*T*_max_), and high-temperature residual carbon content to characterize their thermal stability.

#### 2.1.5 Particle Size Distribution Characteristics

Particle size distribution of the microspheres was determined by using a Malvern Zetasizer Nano ZS90 laser diffraction particle size analyzer. Before testing, microsphere samples were dispersed in an aqueous solution containing 0.1% Tween-20 and subjected to 60 s of ultrasonic treatment to ensure thorough dispersion and eliminate agglomeration. Based on laser diffraction principles, this analysis provides information on the volume particle size distribution of microspheres in the dispersion medium, including characteristic particle sizes (e.g., *D*_10_, *D*_50_, *D*_90_), which are used to evaluate the size uniformity of the microspheres.

#### 2.1.6 Salt Tolerance and Long-Term Stability Evaluation

The long-term stability of microspheres in highly salinity environments was systematically evaluated by using dynamic light scattering. Microspheres were dispersed in simulated B-3 Block formation water (salinity 23,800 mg/L, Ca^2+^+Mg^2+^ 581 mg/L) and hydrated for 1, 30, and 90 days at reservoir temperature (50 °C). A laser particle size analyzer measured the hydrodynamic particle size distribution (including *D*_10_, *D*_50_, and *D*_90_) at each time point to observe temporal trends. By comparing the rate of change in particle size distribution before and after hydration and the shift trend of characteristic particle sizes, the swelling kinetics, structural stability, and salt aging resistance of microspheres in high-salt, high-temperature environments were comprehensively evaluated.

### 2.2 Evaluation of Microsphere Migration and Blocking Performance

#### 2.2.1 Experimental Materials

The core flooding materials used in this experiment primarily include artificial cores, experimental fluids, microsphere samples, and a displacement apparatus.

(1) Experimental Reagents: Experimental fluids comprised simulated formation water and simulated oil. Simulated formation water was prepared based on the ion composition of formation water from the B-3 Block (salinity 23,800 mg/L, with total Ca^2+^+Mg^2+^ ions at 581 mg/L). Simulated oil was a blend of refined kerosene and formation crude oil at a specific ratio, exhibiting a viscosity of 2.8 mPa·s at reservoir temperature (50 °C). Microsphere samples were laboratory-synthesized salt-resistant polymer microspheres (AM/AMPS copolymer). Before use, they were pulverized and sieved, with the 100–200 mesh fraction selected. During experiments, a 1250 mg/L dispersion system was prepared using simulated formation water and magnetically stirred for 30 min to ensure uniform dispersion. All chemical reagents were analytical grade, and deionized water was used for experimental purposes.
(2) Experimental Cores: Artificial cores were used to calculate the resistance coefficient and residual resistance coefficient. The cores had a diameter of 2.4 cm, a length of 6 cm, and a volume of 24.1434 mL.The permeability values were 65.4 × 10^–3^ μm^2^, 146.4 × 10^–3^ μm^2^, 271.8 × 10^–3^ μm^2^, 448.7 × 10^–3^ μm^2^, and 667.4 × 10^–3^ μm^2^.
(3) Experimental Equipment: The displacement apparatus primarily included: an ISCO constant speed, constant-pressure pump (controlling injection flow rates of 0.1–10 mL/min), a core holder (rated for 20 MPa pressure), a pressure sensor (range 0–5 MPa, accuracy ± 0.1% FS), and a data acquisition system.

The experimental flowchart is shown in Figure 1.

**Figure 1:**
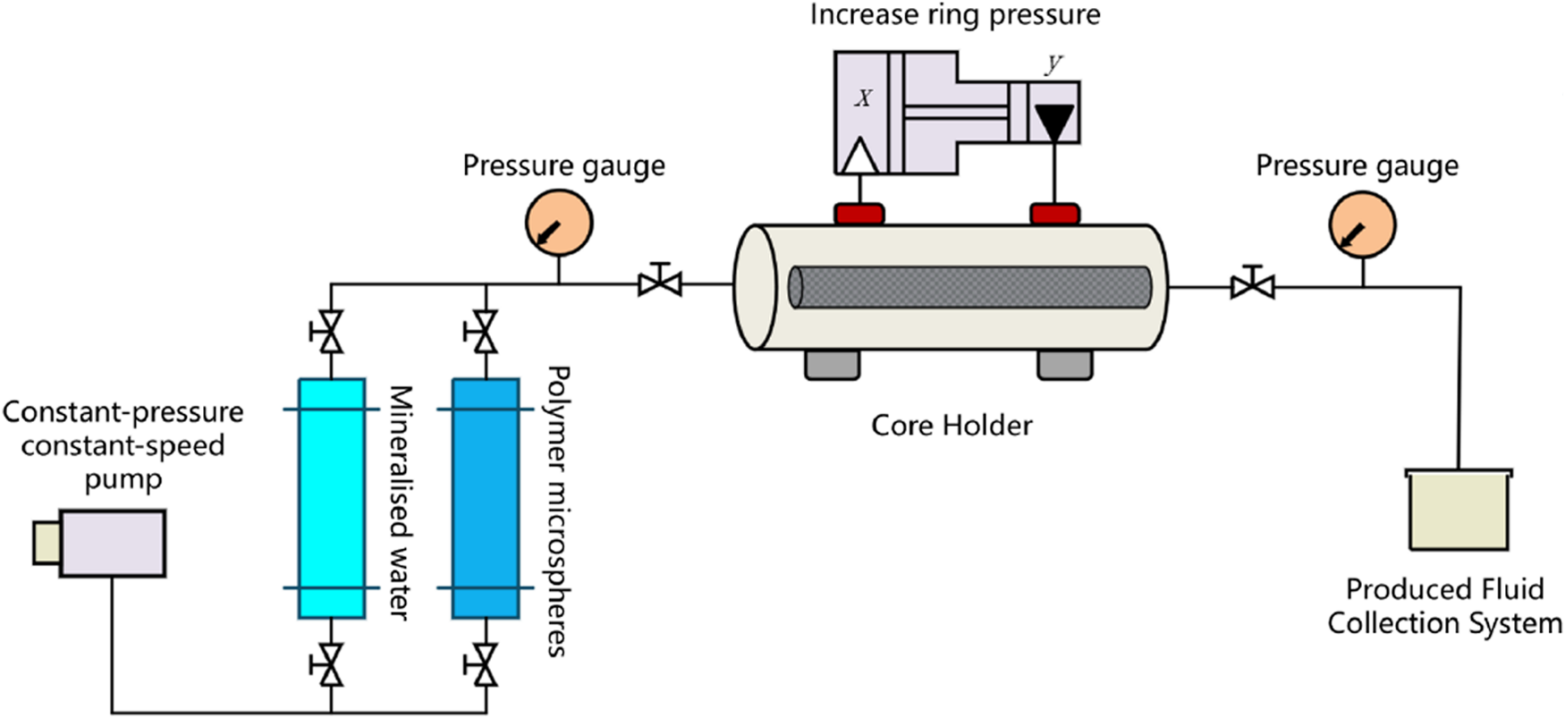
Experimental setup for blocking capacity evaluation.

#### 2.2.2 Experimental Procedure

Single-tube core flow experiments followed standardized operational procedures:

(1) Core pretreatment and fundamental parameter determination: Place artificial cores with permeability of 65.4 × 10^–3^ μm^2^ into core holders. Evacuate pore gas using vacuum. Saturate cores with high-salinity water while simultaneously measuring pore volume (PV) and initial water-phase permeability (*K*_0_).
(2) Microsphere dispersion system injection and pressure monitoring: Using a constant-speed pump at a preset flow rate, inject a 1250 mg/L microsphere dispersion system prepared with high-salinity water into the saturated water core. Record pressure changes at the core inlet using a high-precision pressure sensor throughout the process.
(3) Switching to water flooding and waiting for pressure stabilization: Immediately after microsphere injection completion, switch to high-salinity water flooding (maintaining the same flow rate as microsphere injection). Continue injection until the system pressure enters a plateau phase (minimal pressure variation).
(4) Determine permeability after water flooding stabilization: Once water flooding pressure stabilizes, measure the water-phase permeability of the core (*K*_w_).
(5) Altering the core permeability to 146.4 × 10^–3^ μm^2^, 271.8 × 10^–3^ μm^2^, 448.7 × 10^–3^ μm^2^, and 667.4 × 10^–3^ μm^2^ and repeat the above steps to calculate the Resistance Factor (*RF*), Residual Resistance Factor (*RRF*), and blocking efficiency of polymer microspheres in cores with different permeability values. Analyze the migration and blocking effects of polymer microspheres in cores with varying permeability.

Based on recorded pressure data (Δ*P*_m_: microsphere injection pressure; Δ*P*_w_: initial waterflood pressure) and *K*_0_, *K*_w_, calculate the resistance coefficient and residual resistance coefficient and determine the blocking efficiency.

The calculation formula is as follows:

$$\mathrm{RF}=\frac{K_{o}}{K_{w}}$$

where *K*_0_ is the permeability of the porous medium in its initial state, μm^2^; *K*_w_ is the permeability of the porous medium under specific conditions (e.g., after injection of water-phase or fluid containing plugging agent), μm^2^. *RF* reflects the instantaneous resistance to fluid flow generated during microsphere injection.

$$\mathrm{RRF}=\frac{K_{\mathrm{wo}}}{K_{\mathrm{wf}}}$$

where *K*_wo_ represents the permeability of the porous medium when saturated with water prior to injection of the fluid (e.g., a solution containing the blocking agent), μm^2^. *K*_wf_ represents the permeability of the porous medium when saturated with water after injection of the fluid (e.g., a solution containing the blocking agent) and undergoing a certain seepage process (e.g., displacement, flushing, etc.), μm^2^. *RRF* quantifies the long-term or permanent reduction in core permeability caused by microsphere retention, serving as a core parameter for evaluating the microsphere plugging strength and stability. By analyzing the *RF* and *RRF* values, one can systematically assess the injection capability of the studied salt-resistant polymer microspheres under target reservoir permeability conditions and their plugging performance across different pore-throat scales.

Blocking efficiency (*F*) is a key indicator for evaluating the effectiveness of blocking measures, as defined by the ratio of the reduction in permeability after treatment to the initial permeability. It is typically calculated using the postblocking core permeability (*K*_w_) and the initial permeability (*K*_0_), with the formula:

$$F=\left(1-\frac{K_{w}}{K_{o}}\right)\times 100\%$$

### 2.3 Microsphere Flooding Experiment for Enhanced Oil Recovery

It should be clarified that the core flooding experiments described in Section 2.2 were primarily designed to evaluate the injectivity, resistance factor (*RF*), residual resistance factor (*RRF*), and selective plugging performance of the microsphere system in single cores with different permeability levels. These experiments focused on quantifying the blocking strength and migration characteristics of the microspheres under controlled conditions.

In contrast, the experiments presented in Section 2.3 were conducted to assess the enhanced oil recovery (EOR) performance of the microsphere system under simulated heterogeneous reservoir conditions using parallel core models. Building upon the plugging performance evaluation in Section 2.2, this section investigates how the identified injectivity and selective blocking behavior translate into macroscopic oil recovery improvement, water cut reduction, and displacement efficiency enhancement.

#### 2.3.1 Experimental Materials

The core diameter was 2.5 cm with a length of 30 cm. This experiment employed a dual-tube parallel core system to simulate heterogeneous conditions in the target reservoir. The experimental fluids comprised simulated oil (viscosity of 2.8 mPa·s at 50 °C) and simulated high-salinity water formulated based on the actual formation water ion composition. The displacement agent was a microsphere dispersion system prepared using a self-made salt-resistant polymer microsphere powder (with a series of concentrations). Eight cores with distinct permeability gradients were selected for heterogeneous displacement experiments. The apparent volume, pore volume, porosity, permeability, oil saturation, and permeability gradient for each core are detailed in Table 1.

**Table 1:** Core Parameters for Microsphere Displacement Experiments

**experimental core**	**apparent volume** (mL)	**pore volume** (mL)	**pore volume** (%)	**permeability** (×10^**–3**^ **μm** ^**2**^ **)**	**oil saturation** (%)	**grade difference**
1	147.43	19.68	12.37	59.36	62.1	2.29
2	144.76	19.68	12.38	135.93	63.1	
3	139.58	19.94	13.45	57.92	62.19	4.33
4	147.19	19.94	13.55	250.79	65.19	
5	145.87	19.63	13.64	62.18	64.08	7.08
6	148.35	19.63	13.94	440.23	64.34	
7	147.76	19.87	14.5	64.14	62.17	10.56
8	152.09	19.87	15.2	677.32	63.17	

The displacement experimental setup primarily consisted of a core holder, constant-speed pump, pressure sensor, fraction collector, and data acquisition system to achieve displacement process control and production dynamic monitoring.

#### 2.3.2 Experimental Methods

The evaluation of microsphere-enhanced displacement for enhanced oil recovery (EOR) was conducted through physical simulation displacement experiments:

(1) Core pretreatment and initial state establishment: A dual-tube parallel core simulator replicated heterogeneous reservoir conditions for oil–water displacement. After vacuuming, cores were saturated with high-salinity water. Simulated oil at 50 °C and viscosity 2.8 mPa·s was injected until no water was produced to establish initial oil saturation. Cores were then aged at 50 °C for ≥24 h.
(2) Base water flooding test: Injected high-salinity water at a constant flow rate using a constant-speed pump until the outlet water cut ≥98%. Recorded cumulative oil production and calculated waterflood oil recovery.
(3) Microsphere-enhanced displacement injection: Inject a 1250 mg/L salt-resistant polymer microsphere dispersion system (prepared with high-salinity water) at a volume of 0.15 mg/L
(4) Follow-up Water Flooding Test: Continue injecting high-salinity water at the constant flow rate from the base water flooding test until the outlet water cut reaches ≥98% again.
(5) Data Monitoring and Effect Evaluation: Repeat the above steps while varying injection concentration, injection volume, injection rate, reservoir permeability, and permeability gradient. Calculate water flooding oil recovery, ultimate oil recovery, and enhanced oil recovery. Analyze the influence of different factors on the enhanced oil recovery effect of the microsphere system.

## 3 Results and Discussion

### 3.1 Structure and Properties of Salt-Resistant Polymer Microspheres

#### 3.1.1 Infrared Spectral Characterization of Salt-Resistant Polymer Microspheres

With the use of AM, AMPS, EDTA, and MBA, antisalt polymer microspheres are synthesized. Fourier Transform Infrared Spectroscopy (FTIR) provides conclusive evidence of the chemical structure, as shown in Figure 2. In the tableting method test, the strong absorption peak at 1605.51 cm^–1^ corresponds to the amide I band (C = O stretching vibration), while the characteristic peak at 1448.77 cm^–1^ corresponds to the amide II band (N–H bending vibration), confirming successful polymerization of the AM monomer. The double peaks around 1040 and 1200 cm^–1^ originate from the symmetric and asymmetric stretching vibrations of the −SO_3_^–^ group in AMPS, respectively. The characteristic methylene bridge (−CH_2_) peak at 2922.20 cm^–1^ clearly indicates the participation of the cross-linking agent MBA. Notably, no free acrylamide monomer = N–H stretching vibration peak was detected in the 3100–3500 cm^–1^ region, confirming complete polymerization and corroborating the high thermal stability observed in thermal analysis.

**Figure 2:**
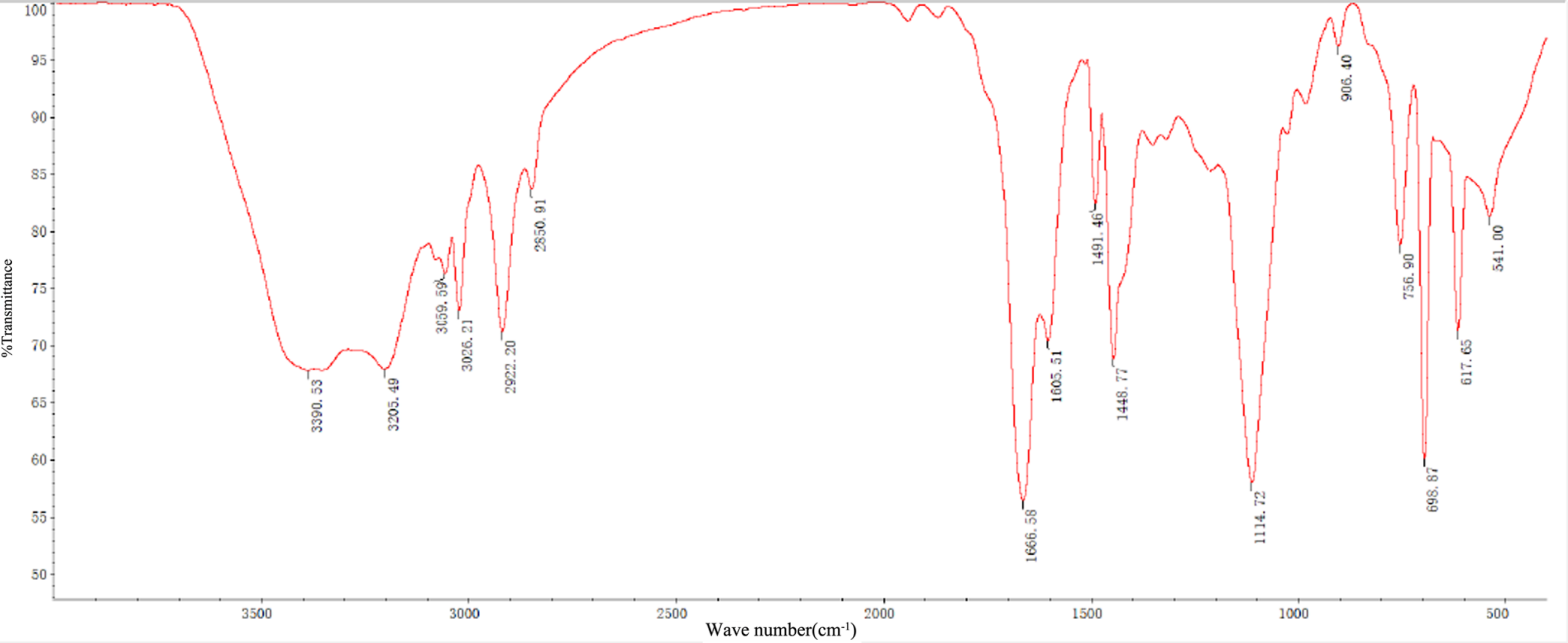
Infrared spectra of a polymer microsphere.

Jin^32^ reveals that the amide group (−CONH_2_) in the molecular structure of the main chain monomer acrylamide (AM) enhances the rigidity of the polymer backbone by forming an intermolecular hydrogen bond network. This hydrogen bond network effectively suppresses the thermal motion of the molecular chains under high-temperature conditions (e.g., 60–80 °C), reducing the risk of segment disentanglement and hydrolysis induced by elevated temperatures. On the other hand, the introduction of 2-acrylamido-2-methylpropanesulfonic acid (AMPS) as a side-chain monomer incorporates strongly hydrophilic sulfonic acid groups (−SO_3_H) bearing significant negative charges. Even in highly salinity or hard ion environments, these groups suppress polymer chain curling and entanglement through electrostatic repulsion, maintaining chain extension to preserve the polymer’s solubility and viscosity. This ensures stability and enhanced oil recovery efficiency in high-salinity conditions.^33^

#### 3.1.2 Thermal Stability Evaluation of Salt-Resistant Polymer Microspheres

To study the thermal stability of the salt-resistant polymer microspheres, thermogravimetric analysis (TGA) was employed to characterize the experimentally synthesized nanoscale polymer microspheres, and the results are shown in Figure 3. The thermogravimetric curve of polymer microsphere particles shows three distinct stages of thermal weight loss. The first stage occurs between 30 and 278 °C, during which intermolecular and intramolecular water evaporation results in a 17% weight loss. The second stage occurs between 278–550 °C, involving the imidization reaction of amide groups and the decomposition of side chains such as AMPS (2-acrylamido-2-methylpropanesulfonic acid), resulting in a weight loss of 79.4%. The third stage occurs above 550 °C, primarily due to polymer carbonization, backbone chain breakage, and decomposition, resulting in a weight loss of 10.8%. The total loss rate across all three stages reaches 90.2%. Overall, significant mass loss in the microspheres was only observed when temperatures exceeded 278 °C, indicating excellent thermal stability. This suggests that the microspheres possess good temperature stability and can be effectively used for reservoir stimulation under high-temperature field conditions without thermal decomposition or significant weight loss.

**Figure 3:**
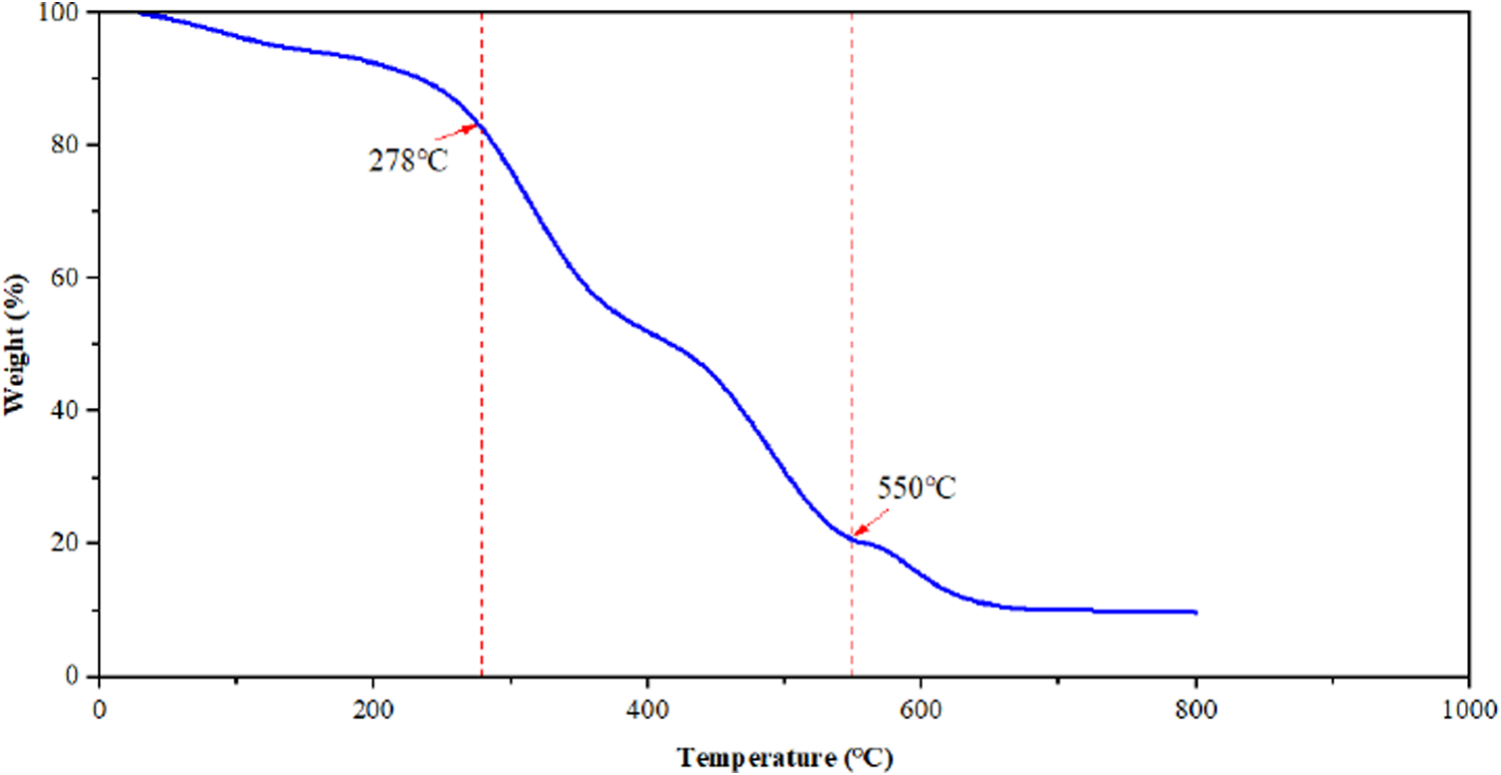
Thermogravimetric curves of polymer microspheres.

The exceptional thermal stability observed can be attributed to the microspheres’ robust molecular architecture. The incorporation of the AMPS monomer is pivotal, as its strong sulfonic acid group confers a high charge density, enhancing intermolecular interactions and forming a rigid network that resists thermal degradation. Furthermore, the amide groups within the polymer backbone undergo cyclization at elevated temperatures (278–550 °C), forming stable imide rings. This structural rearrangement not only consumes energy but also reinforces the polymeric matrix, effectively delaying the onset of massive backbone scission.^34^ Consequently, the microspheres maintain their structural integrity and plugging function in high-temperature reservoirs, ensuring a long-term performance.

#### 3.1.3 Particle Size Distribution Characteristics of Salt-Resistant Polymer Microspheres

The size distribution of the microspheres was characterized to assess their plugging potential. Laser diffraction analysis of the microsphere size distribution is shown in Figure 4. Systematic analysis reveals a unimodal, narrow distribution with a volume-average particle size of 432.8 nm, a *D*_10_ of 328 nm, and a *D*_90_ of 570 nm. This highly uniform distribution is attributed to stable droplet interfaces and balanced shear fields during reverse emulsion polymerization. Abrams^35^ proposed the “1/3” rule theory, suggesting that optimal plugging efficiency occurs when the median particle size of the bridging agent equals or slightly exceeds one-third of the average pore-throat diameter in formation fractures. Hands et al.^36^ proposed the D90 rule, suggesting that optimal particle blocking occurs when 90% of particles in the cumulative distribution curve have diameters smaller than the maximum pore-throat diameter in the formation. The target block’s average pore-throat diameter is 1256.64 nm, and the developed microspheres satisfy the bridging theory. With a D90 of 570 nm, smaller than the maximum pore-throat diameter—the developed microspheres demonstrate excellent blocking performance.

**Figure 4:**
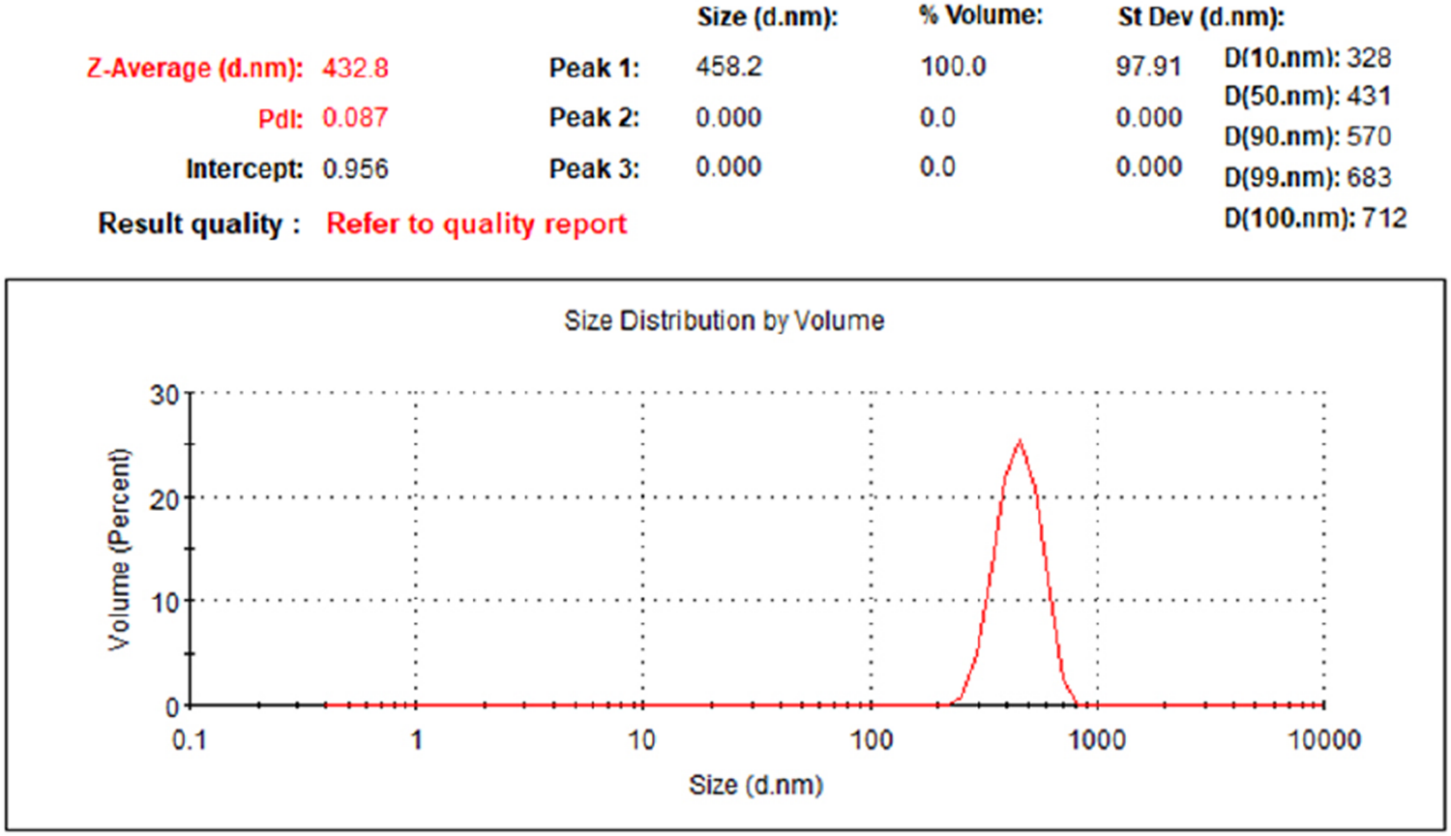
Particle size distribution of polymer microspheres.

The initial average particle size of the microsphere emulsion was 432 nm. In simulated formation water with a salinity of 23,800 mg/L, the initial particle size of the microspheres was 410 nm, indicating that high-salinity water exerts a certain inhibitory effect on the particle size growth of the microspheres. As shown in Figure 5, the particle sizes of the microspheres were similar under different salinity conditions. This phenomenon stems from the incorporation of salt-resistant functional monomer AMPS during polymer microsphere synthesis. The −SO_3_H group in AMPS exhibits tolerance toward ions such as Na^+^, Mg^2+^, Cl^–^, and Ca^2+^ in high-salinity water, thereby preventing the formation of polymer-electrolyte cross-links. Additionally, the presence of amide groups on the microsphere molecular chains reduces the structural influence of the electrolytes. The results demonstrate that the polymer microsphere particles exhibit excellent salt tolerance.

**Figure 5:**
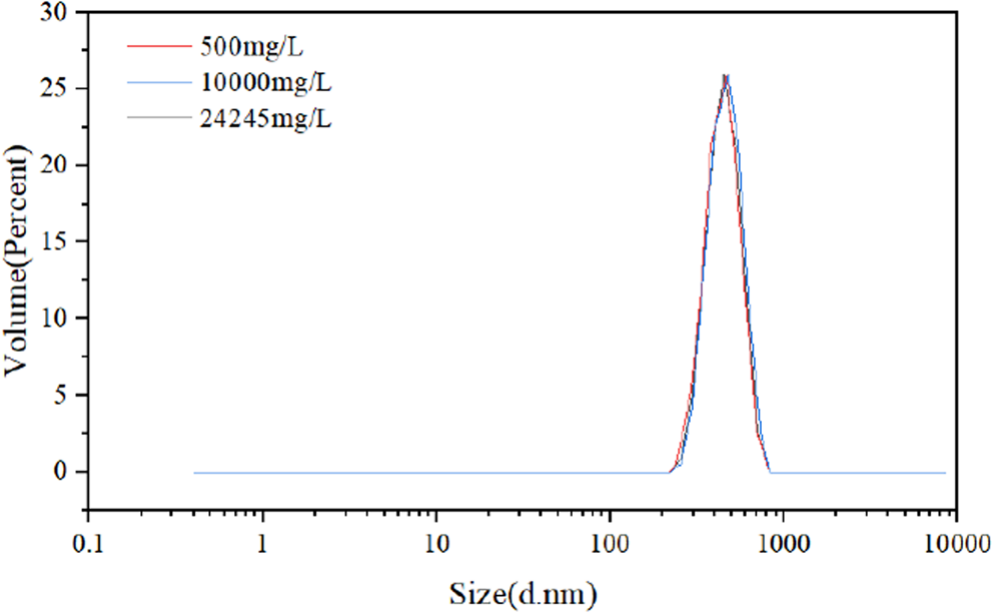
Comparison of microsphere particle size under different mineralization levels.

#### 3.1.4 Microscopic Morphological Characterization of Salt-Resistant Polymer Microspheres

The morphology of polymer microspheres under scanning electron microscopy is shown in Figure 6. The images reveal uniformly spherical particles with a consistent size distribution at the nanoscale. The microspheres exhibit high integrity, featuring smooth, pore-free surfaces, which is beneficial for migration into the reservoir deep. These microspheres act as packers in deep sections of high-permeability reservoirs, mitigating reservoir heterogeneity and enhancing oil recovery. Microscopic observations at various magnifications reveal some agglomeration and adhesion between microspheres. This phenomenon primarily stems from two causes: First, the introduction of self-cross-linking agents disrupts the interpenetrating layers between the core and shell, including chain entanglement or grafting between the core and shell. This leads to monomer migration and the growth of smaller particles, forming relatively continuous structures. Second, the cross-linking groups distributed on the particle surface promote polymer formation.

**Figure 6:**
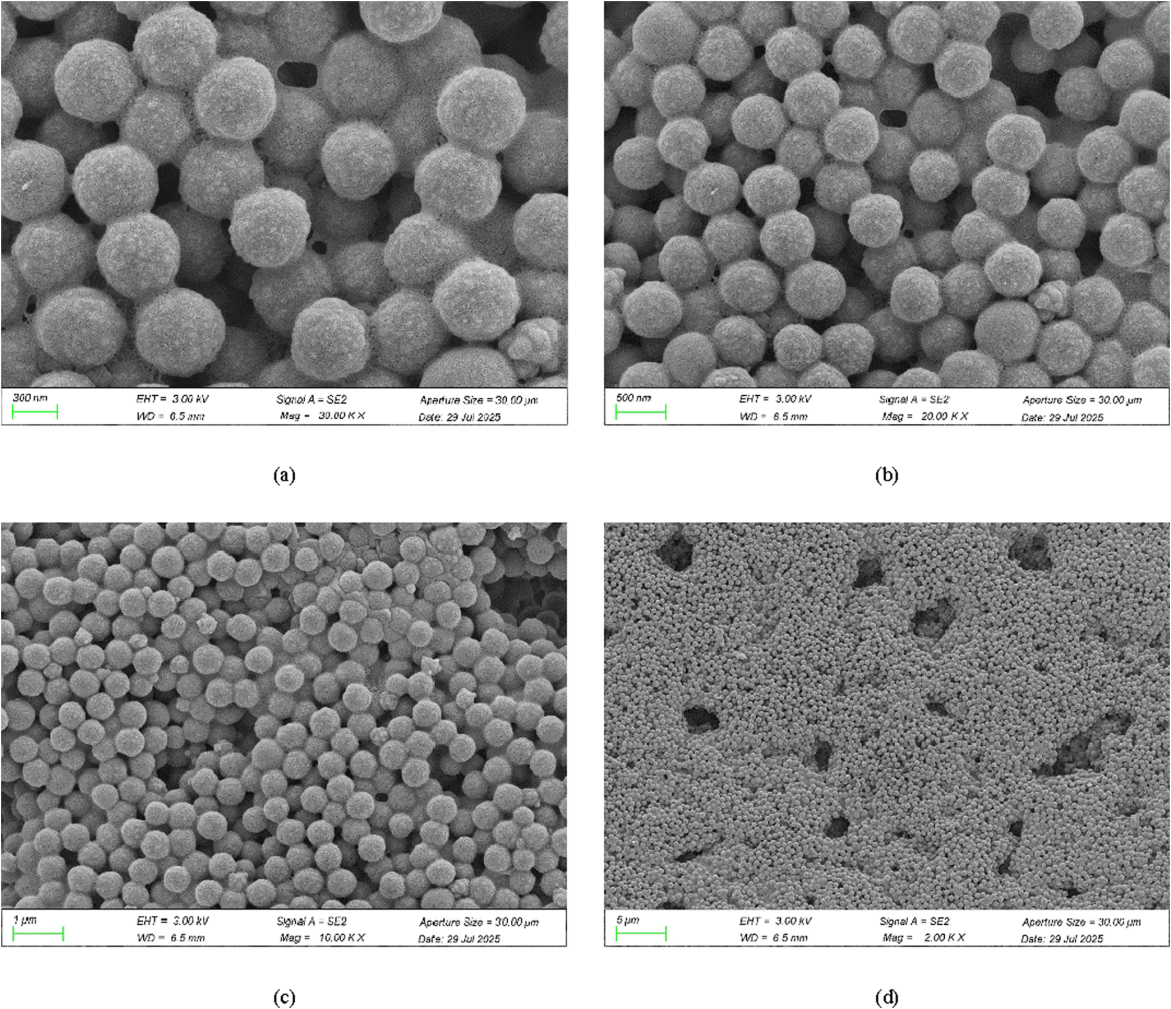
Scanning electron microscopy of polymer microspheres. (a) 300 nm, (b) 500 nm, (c) 1 μm, and (d) 5 μm.

#### 3.1.5 Long-Term Stability Evaluation of Salt-Resistant Polymer Microspheres under High-Salinity Conditions

The long-term stability of polymer microspheres in high-salinity reservoir environments is a critical factor determining their field applicability and operational success. In this study, the long-term thermal, chemical, and mechanical stability of AM/AMPS salt-resistant polymer microspheres was systematically evaluated under simulated reservoir conditions representative of the target block (salinity 23,800 mg/L, Ca^2+^ + Mg^2+^ = 581 mg/L, temperature 50 °C).

#### 3.1.6 Long-Term Thermal Stability

Under simulated formation conditions (50 °C, salinity 23,800 mg/L), the microsphere dispersion was sealed and aged at constant temperature for 90 days, during which particle size evolution and visual morphology were periodically monitored. As shown in Figure 7, the microspheres rapidly absorbed water and reached swelling equilibrium within the first 15 days, after which the particle size remained essentially stable. After 30 days, the variation in D50 relative to the initial value was less than 3%, and after 90 days, the variation remained below 5%. No obvious particle collapse or abnormal aggregation was observed throughout the aging period, indicating that no significant thermal degradation or cross-linked network rupture occurred under long-term isothermal conditions. Combined with the thermogravimetric (TG) analysis presented earlier, which showed no significant thermal decomposition below 278 °C, and considering that the reservoir temperature is only 50 °C, the microsphere system exhibits a substantial thermal safety margin under actual reservoir conditions.

**Figure 7:**
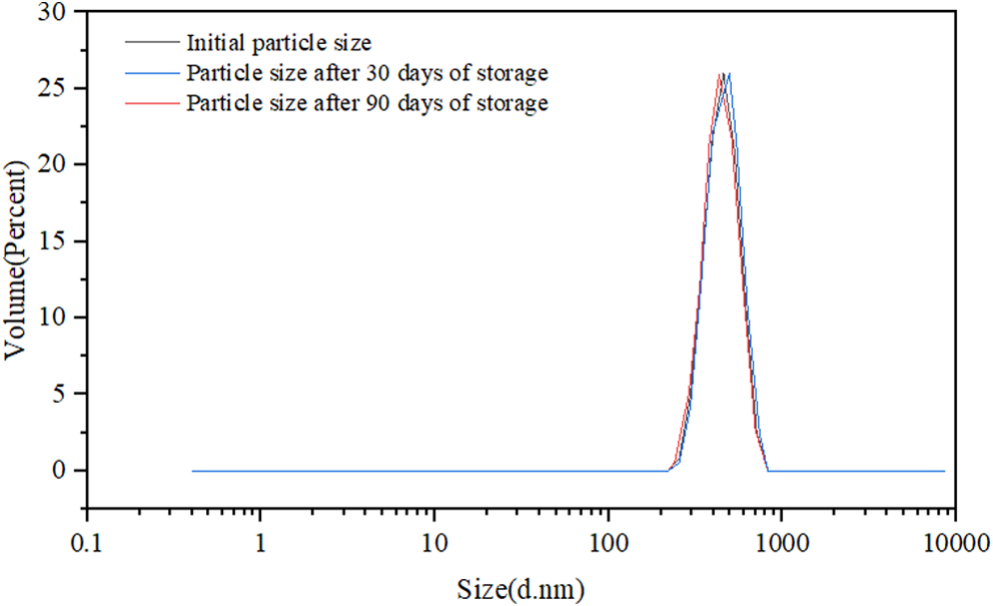
Comparison of microsphere particle sizes at different time points.

#### 3.1.7 Chemical Stability and Salt Resistance during Aging

In high-salinity environments, particularly in the presence of divalent cations such as Ca^2+^ and Mg^2+^, conventional polymer microspheres are prone to charge-shielding-induced chain contraction, ionic bridging that leads to network collapse, and long-term hydrolysis resulting in molecular chain scission. The AM/AMPS copolymer microspheres developed in this study effectively enhance salt resistance through the introduction of sulfonate groups (−SO_3_^–^) from the AMPS monomer. These sulfonate groups possess strong hydration capacity and stable negative charges, which maintain sufficient electrostatic repulsion even under high ionic strength conditions, thereby suppressing chain coiling and structural collapse. During the 90-day aging process, the particle size distribution curves showed no obvious bimodal characteristics or significant broadening, indicating that severe aggregation or structural degradation did not occur. The dispersion system also exhibited no noticeable sedimentation or phase separation, confirming good colloidal stability. Furthermore, a comparison of FTIR spectra before and after aging revealed no significant increase in carboxylate characteristic peaks, suggesting that amide groups did not undergo substantial hydrolysis over the 90-day period and that the chemical structure of the microspheres remained stable.

#### 3.1.8 Mechanical Stability and Shear Resistance

During field injection, microspheres experience multiple mechanical stresses, including pump-induced shear, high-velocity flow scouring, and pore-throat-scale constriction and deformation. Therefore, mechanical stability is a key parameter in evaluating their field applicability and long-term conformance control performance. To systematically assess this property, repeated shear tests were conducted under simulated reservoir conditions using a constant-rate pump at flow rates ranging from 0.1 to 1.0 mL/min. Particle size distributions before and after shear were compared, and the resistance factor (*RF*) and residual resistance factor (*RRF*) were analyzed before and after core flooding. The results showed that after repeated shear cycles, the variation in D50 remained below 4%, with no evidence of significant particle breakage or abnormal aggregation, indicating good structural integrity. Moreover, stable and effective plugging structures could still be formed in low-permeability cores, and even after 90 days of aging, the microsphere system maintained relatively high *RRF* values (>2.5), demonstrating sustained retention capacity and structural durability. These results indicate that the AM/AMPS nanomicrospheres possess excellent elastic deformability, allowing reversible deformation rather than irreversible structural damage during pore-throat constriction and fluid scouring. Consequently, stable bridging structures can be formed in high-permeability channels, enabling persistent flow diversion and deep profile control. This synergistic mechanism of “elastic deformation–stable retention” constitutes the fundamental basis for their long-term effective plugging performance.

#### 3.1.9 Comparison with Conventional Microsphere Systems

Conventional polymer microspheres often exhibit restricted swelling and structural degradation when salinity exceeds 15,000 mg/L or temperature exceeds 60 °C. In contrast, the AM/AMPS microsphere system developed in this study maintained stable particle size and plugging performance under a salinity of 23,800 mg/L and a temperature of 50 °C, with no significant performance decline observed over 90 days. This comparison clearly demonstrates the superior adaptability of the developed system to high-temperature, high-salinity, and low-permeability reservoir conditions.

### 3.2 Injection and Blocking Performance of Salt-Resistant Polymer Microspheres

#### 3.2.1 Injectivity Evaluation

Single-tube core flooding experiments were conducted to systematically evaluate the injection capability and throat blocking effectiveness of the developed salt-resistant polymer microsphere system in artificial cores with varying permeability. Experimental results revealed the key flow and blocking characteristics of this system under simulated high-salinity, low-permeability reservoir conditions.

Experimental results indicate that under conditions of B-3B-3 Block formation water (salinity 23,800 mg/L), a microsphere dispersion system at 1250 mg/L concentration can be successfully injected into rock cores with permeability ranging from 50 × 10^–3^ μm^2^ to 700 × 10^–3^ μm^2^, with no significant near-wellbore blockage observed. The relationship between the displacement pressure differential and injection volume is shown in Figure 8. Throughout the injection process, pressure rose steadily and remained controllable, indicating excellent dispersion stability and migration capability of microsphere particles within the pore network. Their moderate initial particle size structure and elasticity enable deformation to adapt to pore channels of varying scales, a critical prerequisite for ensuring deep penetration into the reservoir for displacement enhancement.

**Figure 8:**
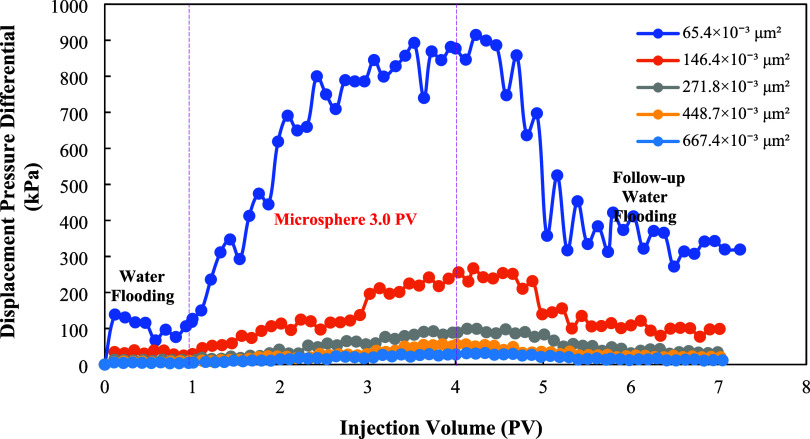
Relationship between displacement pressure differential and injection volume.

As shown in Figure 8, during the microsphere injection phase, the injection pressure increased as rock permeability decreased. Compared to the water flooding phase, injecting the microsphere solution effectively increased the viscosity of the water phase, thereby raising flow resistance during displacement. Consequently, the pressure rise was more pronounced. During the subsequent water injection phase, lower permeability correlated with faster pressure rise and higher breakthrough pressure. Consequently, the final steady-state pressure was relatively high. It was observed that after polymer microsphere injection into sand-packed pipes with varying permeabilities, the injection pressure for secondary water injection exceeded that of primary water injection, indicating the blocking effect of polymer microspheres.

Although no significant injectivity issues or plugging were observed under the experimental conditions (injection rate of 0.3 mL/min, concentration of 1250 mg/L, and slug size of 1.0 PV), it is essential to assess the maximum allowable injection pressure and operational risk limits for field applications. Based on the estimated formation fracture pressure gradient (approximately 0.016–0.018 MPa/m), the fracture pressure at the target reservoir depth (∼1500 m) is approximately 24–27 MPa. The highest injection pressure recorded during core flooding experiments occurred in low-permeability cores (∼60 × 10^–3^ μm^2^), with a peak value of around 2.8 MPa—well below the lower bound of the fracture pressure. This indicates that the current injection parameters are operationally safe.

However, in practical field implementation, excessively high injection rates or microsphere concentrations may lead to rapid pressure buildup near the wellbore, potentially increasing the risk of formation fracture or microfracture propagation. Therefore, it is recommended to set an upper injection pressure limit at 80% of the formation fracture pressure (approximately 19–22 MPa) and to implement real-time pressure monitoring with feedback control. If abnormal pressure increases are detected (e.g., exceeding 15 MPa or a continuous rise of more than 30%), injection parameters should be promptly adjusted—such as reducing the injection rate or temporarily suspending injection, to prevent formation damage.

#### 3.2.2 Blocking Performance and Selectivity

The microsphere system exhibits significant blocking capability, manifested by a high resistance factor (*RF*) and residual resistance factor (*RRF*), as shown in Figure 9. In cores with permeability approximately 60 × 10^–3^ μm^2^ (close to the average reservoir permeability of 63.2 × 10^–3^ μm^2^ in the B-3 Block), the *RF* value reached over 6, while the *RRF* value exceeded 2.5. High *RF* values indicate that microspheres rapidly generate significant flow resistance during injection, forcing the injected fluid to change direction. High *RRF* values indicate that after being retained in the pore throat, the microspheres cause a significant and relatively persistent reduction in core permeability, demonstrating the formation of an effective physical plug.

**Figure 9:**
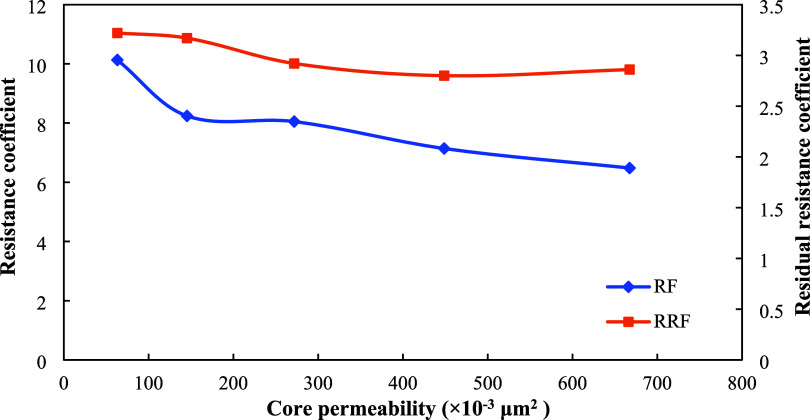
Relationship between *RF*, *RRF*, and core permeability.

The blocking efficiency is shown in Table 2. As the core permeability increases, although the *RF* and *RRF* decrease, the blocking efficiency gradually improves. Notably, when permeability exceeds 448.7 × 10^–3^ μm^2^, the blocking efficiency reaches over 90%, indicating superior plugging performance in high-permeability zones and demonstrating a selective blocking capability (i.e., “blocking large pores while sparing small ones”). This suggests that the microsphere system can form effective flow barriers, even in low-permeability pore throats. The significant increase in resistance factor (*RF*) and residual resistance factor (*RRF*), combined with the high plugging efficiency observed in high-permeability cores (Table 2), suggests that the microspheres undergo moderate deformation at narrow pore-throat entrances and achieve physical plugging via a bridging mechanism, thereby altering the flow paths of subsequent injected fluids. As a result, fluids are diverted into previously unswept low-permeability oil-bearing zones, significantly improving the reservoir sweep efficiency. These results demonstrate the excellent adaptability of this salt-resistant polymer microsphere system under low-permeability conditions, highlighting its importance for enhancing oil recovery in such low-permeability heterogeneous reservoirs in the B-3 Block.

**Table 2:** Influence of Different Permeability on the Plugging Performance of Microsphere System

**permeability** (×10^**–3**^ **μm** ^**2**^ **)**	***RF***	***RRF***	**plugging efficiency**
63.2	10.13	3.22	78.21
145.4	8.24	3.17	81.53
271.8	8.05	2.92	83.73
448.7	7.14	2.8	90.78
667.4	6.48	2.86	95.86

The plugging efficiency (*F*) presented in Table 2 is a key indicator for evaluating the blocking performance of the microsphere system. It is defined as the ratio of the permeability reduction after treatment to the initial permeability, calculated as *F* = (1–*K*_0_/*K*_w_) × 100%, where *K*_0_ is the initial water-phase permeability of the core, and *K*_w_ is the water-phase permeability measured after microsphere injection and subsequent water flooding stabilization. A higher value indicates a more effective physical plugging formed by microspheres within the pore throats.

### 3.3 Enhanced Oil Recovery Effect of Salt-Resistant Polymer Microspheres

To comprehensively evaluate the enhanced oil recovery (EOR) potential and key influencing factors of the developed salt-resistant polymer microsphere system under simulated high-salinity, low-permeability reservoir conditions in the B-3 Block, this study conducted systematic physical simulation oil displacement experiments. The experiments aimed to quantitatively reveal the influence of operational parameters (microsphere concentration, injection rate, and injection volume) and reservoir geological parameters (permeability and permeability variation) on the oil recovery enhancement achieved by microsphere displacement.

#### 3.3.1 Effect of Microsphere Concentration on Displacement Efficiency

As shown in Figure 10a, the oil recovery during the water flooding phase initially increased rapidly before plateauing. The terminal oil recovery for all microsphere concentration systems was approximately 45%. This phenomenon indicates significant water channeling during the later stages of water flooding, leading to the reduction of oil displacement efficiency. Upon transitioning to the microsphere-enhanced displacement phase, the oil recovery markedly increased again. Furthermore, higher microsphere concentrations resulted in greater oil production increases. High-concentration microspheres (e.g., 1250 and 1500 mg/L) form effective blockages in dominant flow channels, redirecting injected water to unexploited zones and mobilizing more residual oil.

**Figure 10:**
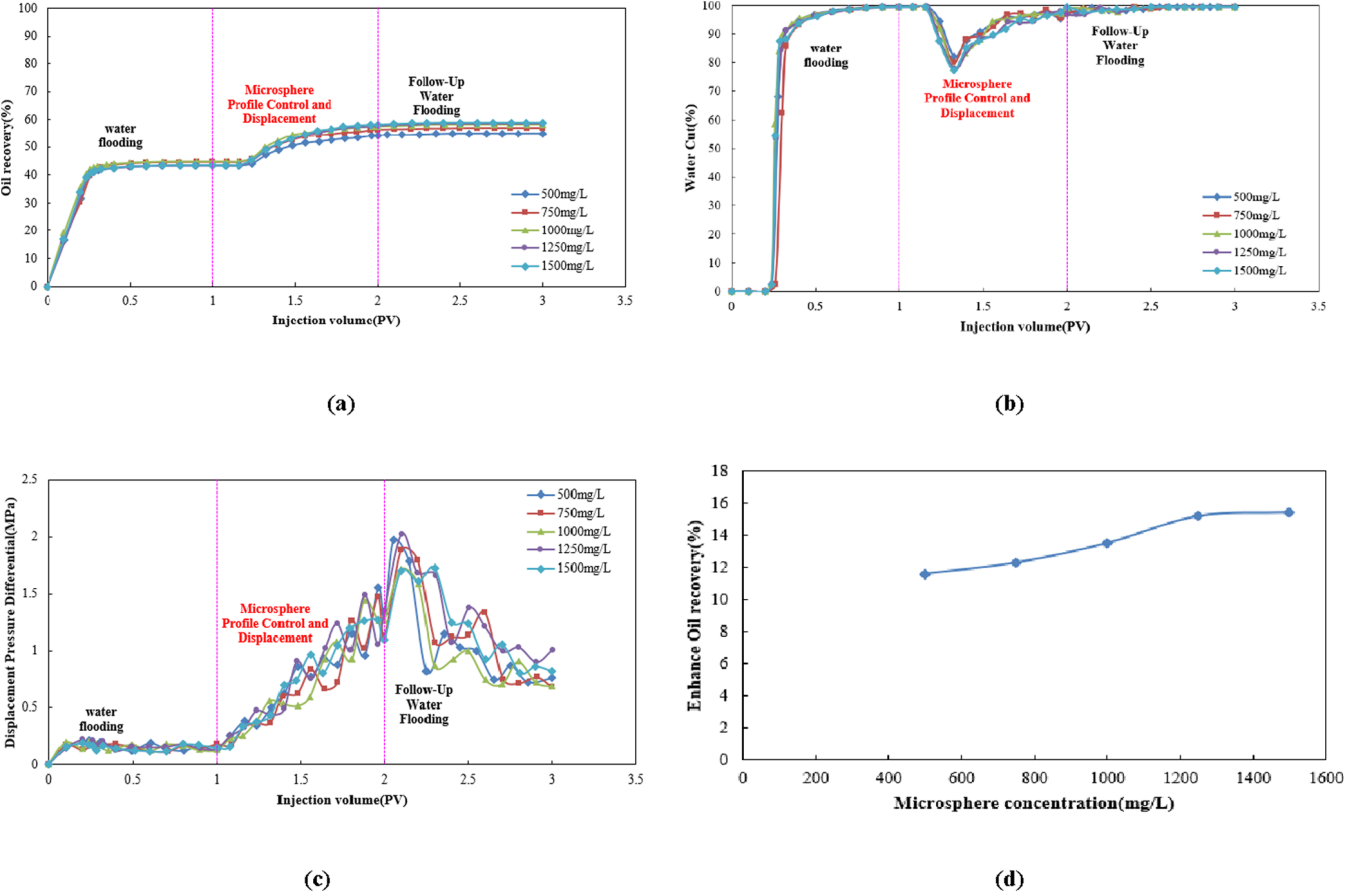
(a) Dynamic oil recovery curve, (b) dynamic water cut curve, (c) dynamic displacement pressure differential curve, and (d) comparison of enhanced oil recovery.

Figure 10b shows that during the water flooding stage, the water cut rapidly increased to nearly 100%, reflecting severe water channeling issues. Upon transitioning to the microsphere-enhanced flooding stage, the water cut exhibited a pronounced “V-shaped” decline, with higher-concentration microsphere systems achieving greater reductions. This indicates their superior effectiveness in curbing ineffective water circulation and improving the swept volume, lowering the water cut to below 70%. During the subsequent water flooding stage, the water cut gradually recovered but stabilized at a significantly lower level than during the water flooding stage (approximately 98 vs. ≈100%), and decreased with increasing microsphere concentration.

Figure 10c shows that the displacement pressure differential during the water flooding stage was low and stable; during the microsphere-enhanced flooding stage, the displacement pressure differential rose rapidly, fluctuated after reaching a peak, and then gradually decreased during the subsequent water flooding stage. The peak displacement pressure differential and fluctuation patterns differed among the microsphere concentrations.

Figure 10d indicates that the enhanced oil recovery increases progressively as microsphere concentration rises from 500 to 1500 mg/L, demonstrating that higher microsphere concentrations yield more significant oil recovery improvements, with enhancement ranging from 12 to 16%. A distinct inflection point was observed at a concentration of 1250 mg/L, corresponding to an oil recovery of 15.22%. This concentration is therefore recommended as the optimal injection concentration.

#### 3.3.2 Effect of Microsphere Injection Volume on Displacement Efficiency

As shown in Figure 11a, during the water flooding stage (injection volume 0–0.5 PV), the oil recovery rose rapidly before leveling off, with minimal differences among injection volume groups. During the microsphere displacement phase (injection volume 0.5–2.5 PV), oil recovery significantly increased, with larger injection volumes (e.g., 1.50 PV group) yielding more pronounced oil gain. This indicates that higher microsphere injection volumes enhance the blocking effect, enabling more effective displacement front migration toward low-permeability zones. During the subsequent water flooding stage (injection volume >2.5 PV), the oil recovery continued to rise slowly and stabilized, with the high-injection-volume group achieving a significantly higher ultimate oil recovery.

**Figure 11:**
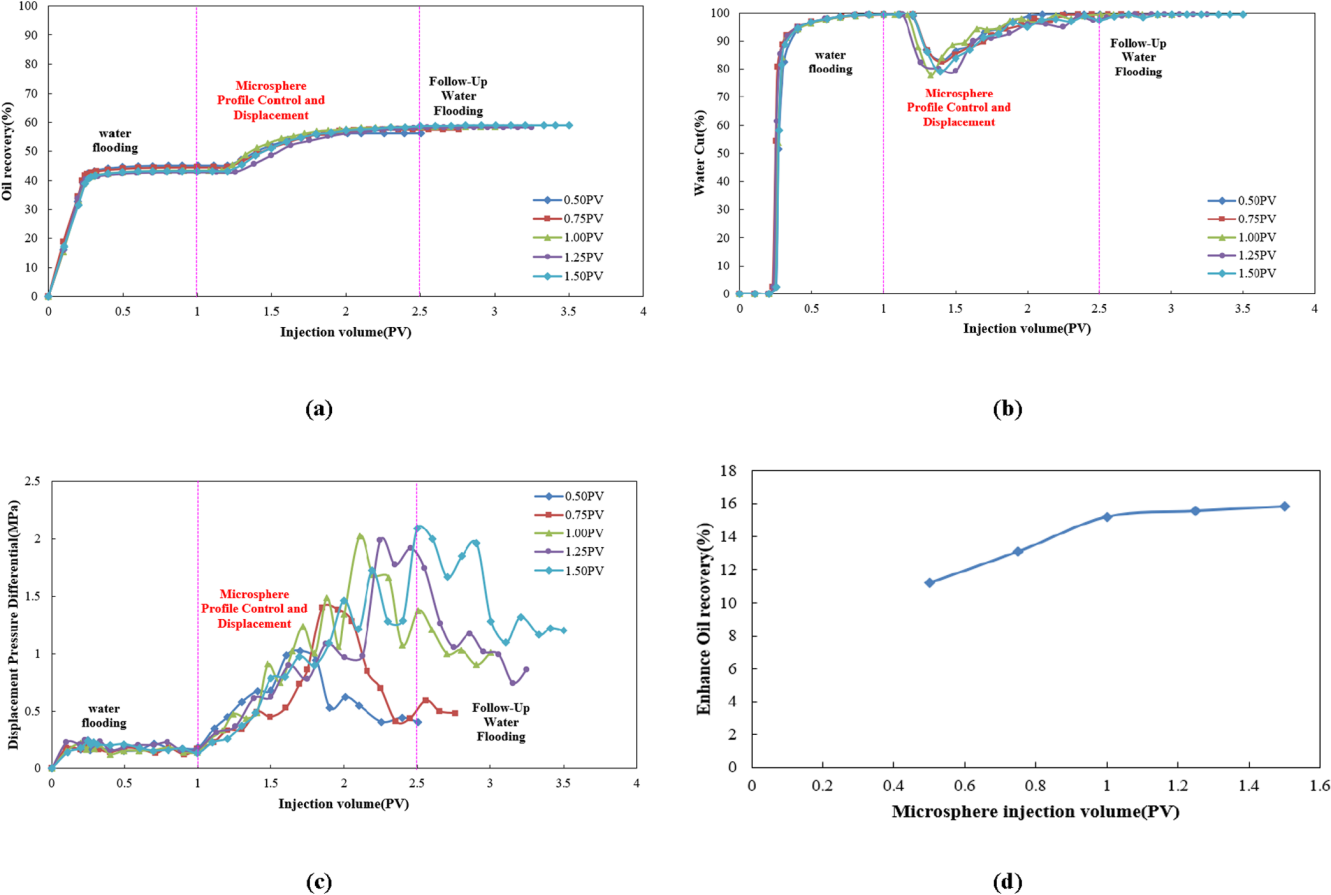
(a) Dynamic oil recovery curve, (b) dynamic water cut curve, (c) dynamic displacement pressure differential curve, and (d) comparison of enhanced oil recovery.

Figure 11b shows an opposite trend to oil recovery. During the water flooding phase, the water cut rapidly increased to nearly 100%. In contrast, the microsphere-assisted flooding phase exhibited a pronounced “V-shaped” decline in water cut. Higher injection volumes resulted in more significant reductions, lowering the water cut to 77–83%. This indicates the increased participation of microspheres in blocking dominant flow pathways. Although the water cut rebounded during the subsequent water flooding phase, it remained significantly lower than the water flooding phase after stabilization, verifying the persistent effectiveness of the high-injection-volume microsphere system in mitigating water breakthrough and enhancing the water flooding efficiency.

Figure 11c shows that the displacement pressure differential during the water flooding stage was low and stable; during the microsphere-enhanced water flooding stage, the displacement pressure differential rapidly rose to a peak before fluctuating; and during the subsequent water flooding stage, the displacement pressure differential gradually decreased. The peak displacement pressure differentials and fluctuation patterns varied among different microsphere concentrations.

Figure 11d indicates that increasing microsphere injection volume from 0.5 to 1.5 PV enhances oil recovery by 11–16%. As injection volume increases, the oil recovery enhancement first increases and then levels off. An inflection point in oil recovery was observed when the injection volume reached 1.0 PV. Therefore, this volume is recommended as the optimal injection volume.

#### 3.3.3 Effect of Microsphere Injection Rate on Displacement Efficiency

Figure 12a analysis indicates that during the water flooding stage, oil recovery for all injection rate groups rose rapidly before stabilizing. In the microsphere flooding stage, oil recovery increased again, with the most significant oil production enhancement observed at an injection rate of 0.1 mL/min. This demonstrates that moderate injection rates can enhance microsphere accumulation and blocking effects within high-permeability channels while maintaining migration capability. During the subsequent water flooding stage (>2.0 PV), oil recovery increased, gradually slowed, and the differences in ultimate oil recovery among the various injection rate groups narrowed.

**Figure 12:**
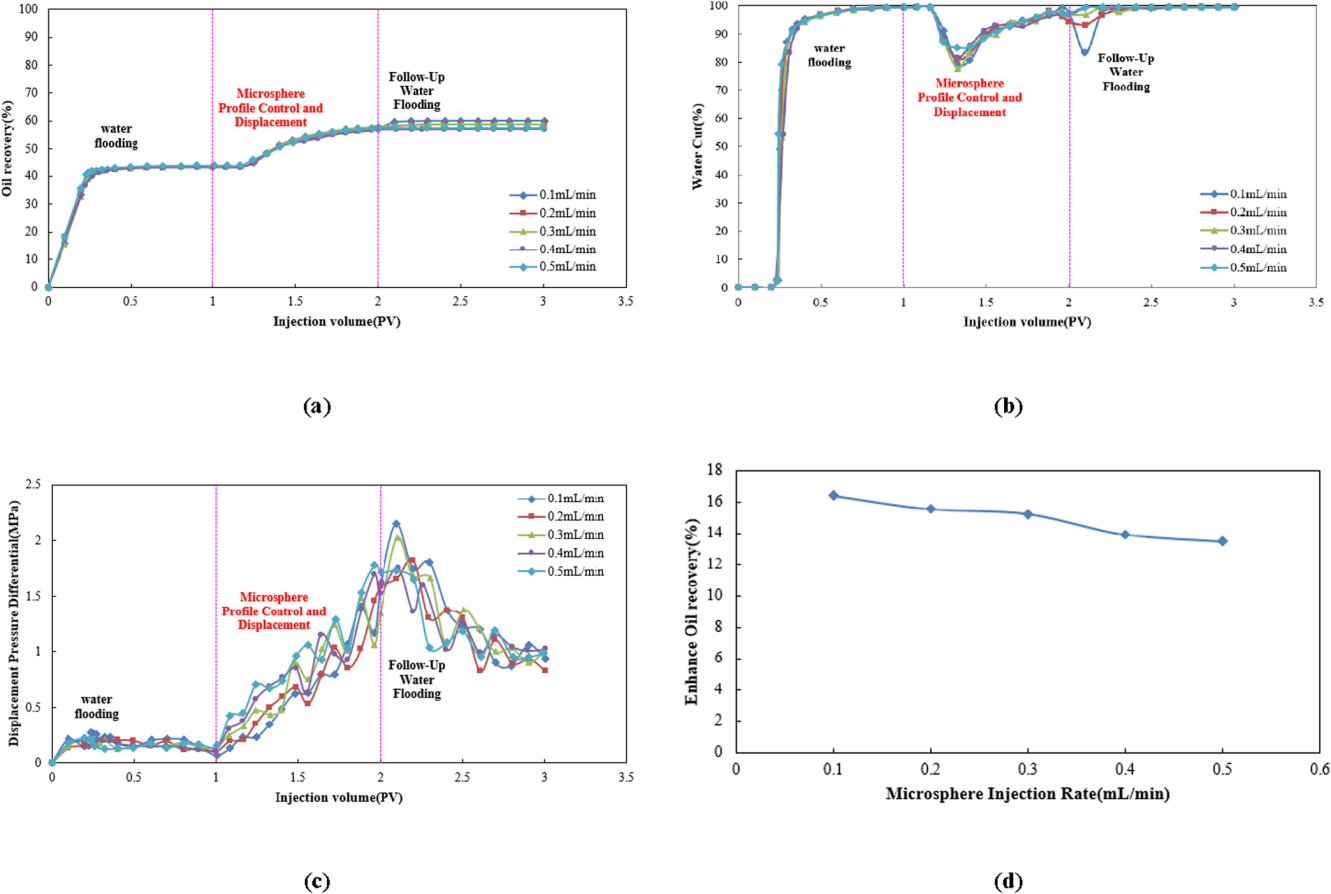
(a) Dynamic oil recovery curve, (b) dynamic water cut curve, (c) dynamic displacement pressure differential curve, and (d) comparison of enhanced oil recovery.

Figure 12b shows the curves for the water flooding, microsphere adjustment flooding, and subsequent water flooding phases. During the water flooding phase, the water cut rapidly increased to nearly 100%. In the microsphere adjustment flooding phase, the water cut decreased significantly, demonstrating the microspheres’ ability to block high-permeability channels. In the subsequent water flooding phase, the water cut gradually rebounded and stabilized. The magnitude of the change in each phase varied with different injection rates, with the water cut ultimately decreasing to 77–81%.

Figure 12c covers three phases: water flooding, microsphere-enhanced flooding, and subsequent water flooding. During water flooding, the displacement pressure difference remained low and stable. In the microsphere-enhanced flooding phase, the displacement pressure difference rapidly increased, fluctuated after reaching its peak, and gradually decreased during subsequent water flooding. The displacement pressure difference trends are consistent across different injection rates (0.1, 0.2, 0.3, 0.4, and 0.5 mL/min), but the peak magnitude and fluctuation intensity vary.

Figure 12d shows that increasing microsphere injection rate from 0.1 to 0.5 mL/min enhances oil recovery by 14–17%. As the injection rate increases, the oil recovery enhancement first decreases slowly and then rapidly. This indicates that higher injection rates yield weaker oil recovery enhancement effects.

#### 3.3.4 Effect of Reservoir Permeability on Displacement Efficiency

Permeability significantly influences the microsphere flooding performance. Figure 13a indicates that high-permeability reservoirs (e.g., 666.3 × 10^–3^ μm^2^) exhibit faster initial oil recovery increases during water flooding but reach a plateau sooner, suggesting a higher water breakthrough risk. During microsphere flooding, medium-to-high permeability reservoirs (262.6–666.3 × 10^–3^ μm^2^) achieved the greatest oil recovery increase, indicating more effective microsphere blocking in high-permeability channels. In subsequent water flooding, ultimate oil recovery slightly improved with increasing permeability, but the gap gradually narrowed, demonstrating microsphere flooding’s excellent long-term adaptability across reservoirs with varying permeabilities.

**Figure 13:**
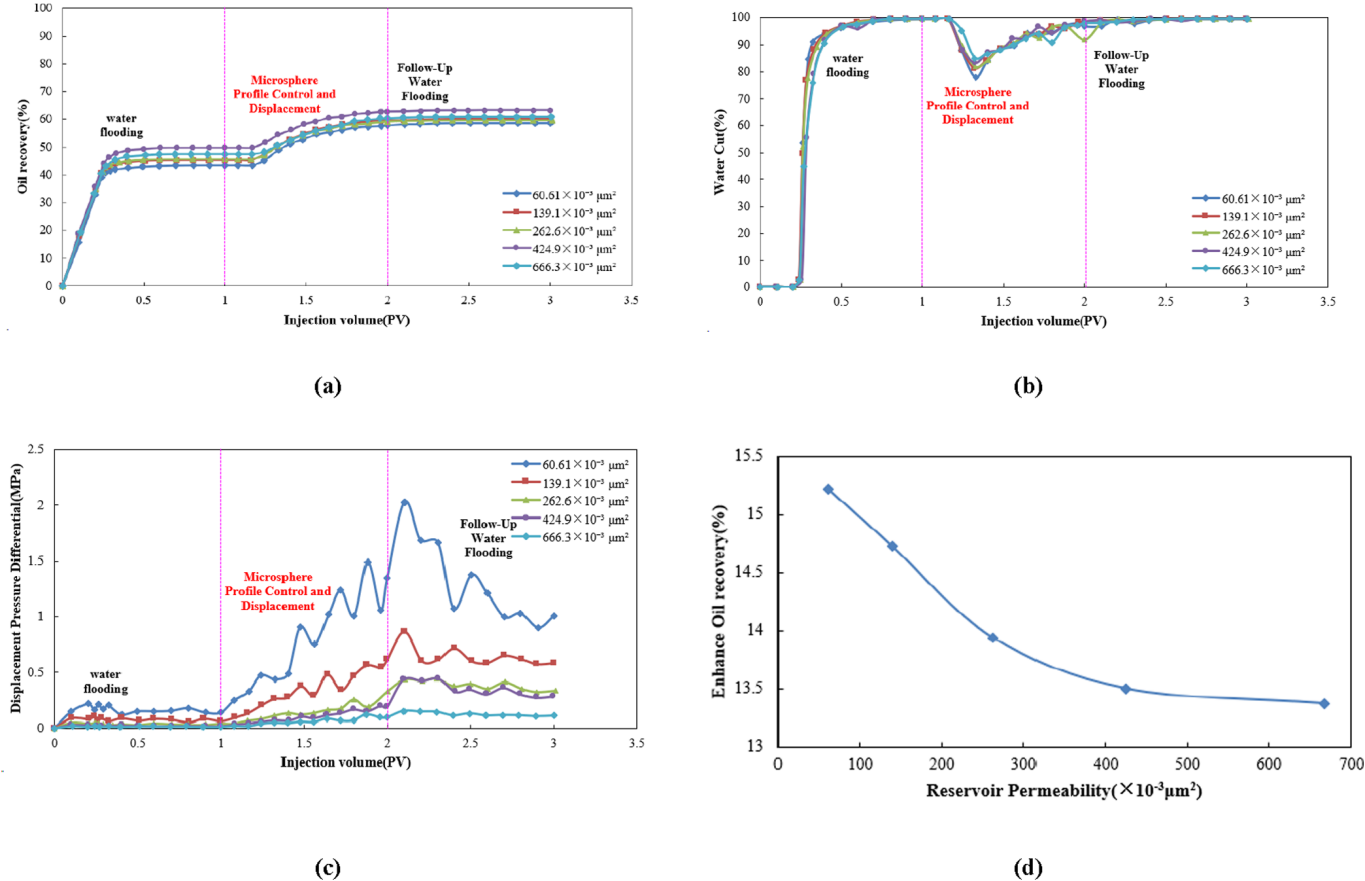
(a) Dynamic oil recovery curve, (b) dynamic water cut curve, (c) dynamic displacement pressure differential curve, and (d) comparison of enhanced oil recovery.

Figure 13b shows that during the water flooding stage, higher permeability resulted in faster water cut increases to 100% and more severe water migration. During the microsphere flooding stage, high-permeability reservoirs exhibited greater water cut reductions, demonstrating the microspheres’ efficient blocking capability in dominant channels, with water cut decreasing to 77–85%. Although WOD slightly rebounded in the subsequent water flooding phase, it remained significantly lower than during water flooding, indicating that microsphere flooding substantially mitigates water breakthrough in high-permeability reservoirs and enhances oil recovery efficiency.

Figure 13c illustrates displacement pressure differential variations across different reservoir permeabilities. The water flooding stage exhibits a low and stable displacement pressure differential. During microsphere-assisted flooding, the displacement pressure differential rapidly rises to a peak before fluctuating. Subsequently, the displacement pressure differential gradually decreases during the water flooding stage. Significant differences exist in peak displacement pressure differentials and fluctuation levels across varying permeabilities, with lower-permeability reservoirs exhibiting higher peak displacement pressure differentials.

Figure 13d shows an enhanced oil recovery (EOR) of 13–16%. As reservoir permeability increases from 60.61 × 10^–3^ μm^2^ to 666.3 × 10^–3^ μm^2^, the oil recovery enhancement gradually decreases. This indicates that higher reservoir permeability weakens the oil recovery enhancement effect of microsphere-assisted flooding.

#### 3.3.5 Effect of Permeability Gradient on Displacement Efficiency

Figure 14a shows that during the water flooding phase, oil recovery differs little among reservoirs with varying permeability differentials. However, during the microsphere-enhanced flooding phase (0.5–2 PV), reservoirs with smaller permeability differentials (e.g., 1.0, 2.3) exhibit greater oil recovery increases. This indicates that microspheres enable more uniform fluid wetting in homogeneous reservoirs, thereby sustainably enhancing oil recovery. In the subsequent water flooding stage, reservoirs with smaller permeability differentials (e.g., 2.3) ultimately achieved the highest ultimate oil recovery (approaching 60%), indicating stronger long-term oil enhancement potential.

**Figure 14:**
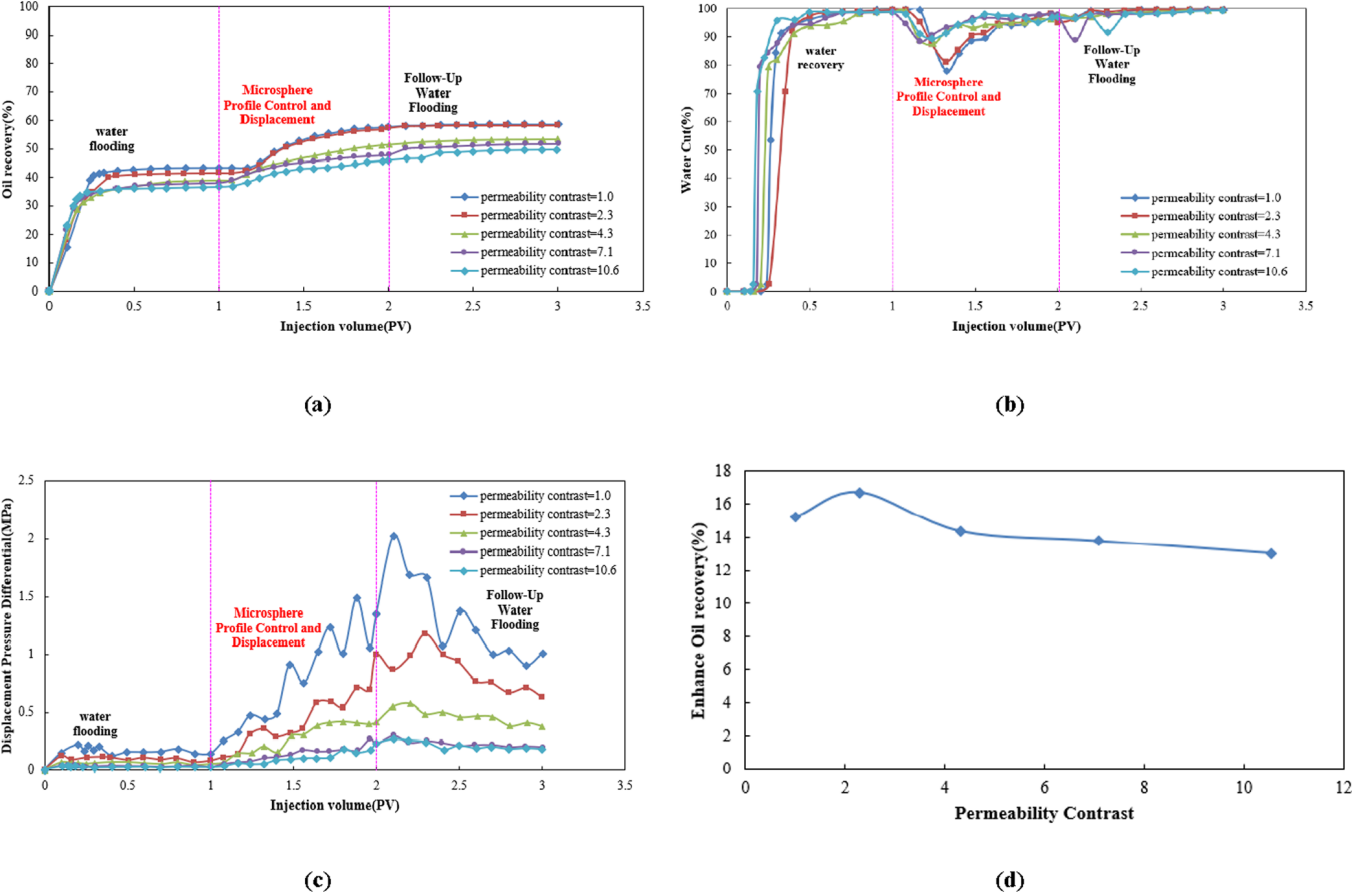
(a) Dynamic oil recovery curve, (b) dynamic water cut curve, (c) dynamic displacement pressure differential curve, and (d) comparison of enhanced oil recovery.

Figure 14b shows that during water flooding, larger permeability differentials (e.g., 10.6) result in faster water cut increases and more severe water breakthrough. Conversely, larger differentials during the microsphere flooding phase lead to more pronounced water cut reductions, indicating that in heterogeneous reservoirs, microspheres effectively seal high-permeability channels and block water breakthrough pathways, lowering water cut to 77–88%. During the subsequent water flooding stage, the water cut slightly increases with higher gradient values but remains significantly lower than during the water flooding stage.

Figure 14c encompasses the water flooding, microsphere-assisted flooding, and subsequent water flooding phases. The water flooding phase exhibits a low and stable displacement pressure differential. During the microsphere-assisted flooding phase, the displacement pressure differential rises rapidly, with distinct variations in peak values and fluctuations across different permeability differentials (1.0, 2.3, 4.3, 7.1, and 10.6). In the subsequent water flooding phase, the displacement pressure differential shows varying degrees of fluctuation or decline.

As shown in Figure 14d, the enhanced oil recovery (EOR) initially increases with injection volume, reaching a peak enhancement of approximately 17%, followed by a gradual decline. This trend indicates the existence of an optimal injection volume (approximately 1.0 PV under the tested conditions). The underlying rationale is that at lower injection volumes, the number of microspheres retained in high-permeability channels is insufficient to form effective plugging, limiting sweep efficiency improvement. Conversely, excessive injection leads to over-retention and potential pore-throat blockage, particularly in low-permeability zones, which may impair injectivity and reduce overall displacement efficiency. Therefore, the optimal injection volume represents a balance between sufficient plugging for flow diversion and controlled retention to avoid formation damage, maximizing the synergistic effect of microsphere-assisted enhanced oil recovery.

Based on the comprehensive analysis of the aforementioned factors, the optimal injection parameters were determined as a concentration of 1250 mg/L, an injection volume of 1.0 PV, and an injection rate of 0.3 mL/min. Under this optimum scheme, an incremental oil recovery of 16.86% was achieved with a water cutoff of 75.67%. Furthermore, this parameter combination demonstrated robust performance and good adaptability, effectively enabling in-depth profile control and displacement across cores with varying permeabilities and heterogeneities.

Sensitivity analysis indicates that the microsphere flooding system exhibits a moderate dependence on injection parameters within the investigated ranges. Oil recovery does not show abrupt deterioration when the concentration, injection rate, or slug size deviates moderately from the identified optimum values. Specifically, within a concentration range of 1000–1500 mg/L, an injection rate of 0.2–0.4 mL/min, and a slug size between approximately 0.8 and 1.2 PV, the incremental oil recovery remains relatively stable, with only gradual variations observed. This suggests the existence of a reasonably broad operational window, indicating that minor fluctuations in field injection conditions are unlikely to cause significant performance loss. Such tolerance enhances operational flexibility and supports the practical applicability of the microsphere flooding strategy under real reservoir conditions.

Economic evaluation is essential for assessing the feasibility of a field-scale implementation. The proposed nanosphere flooding system operates at a relatively moderate concentration (1250 mg/L) and a limited slug size (1.0 PV), resulting in controlled chemical consumption compared with conventional high-viscosity polymer flooding, which typically requires continuous injection at higher concentrations. Based on a preliminary economic calculation, the chemical cost of the nanosphere system is approximately USD 45–55 per barrel of incremental oil, while conventional polymer flooding usually costs USD 80–100 per barrel of incremental oil under similar reservoir conditions. The incremental oil recovery of approximately 15–17% under representative reservoir conditions indicates a favorable potential return relative to chemical dosage. From an operational perspective, the microsphere dispersion can be prepared directly using high-salinity formation water without complex pretreatment, and injection can be performed using existing water flooding facilities, minimizing additional surface infrastructure requirements. Core-scale experiments demonstrate stable injectivity without excessive pressure buildup, suggesting a manageable field construction risk. Compared with alternative conformance control technologies such as in situ cross-linked gels, conventional polymer flooding, or foam systems, the nanosphere system provides a balanced combination of injectivity, selective plugging capability, salt tolerance, and deep migration performance, while reducing risks of premature gelation, near-wellbore blockage, or severe shear degradation. These characteristics indicate that the proposed system possesses competitive technical and economic feasibility for application in high-salinity, low-permeability reservoirs.

In addition to the physical plugging mechanism, the AM/AMPS nanosphere system may indirectly influence the oil displacement efficiency through interfacial interactions. The amide and sulfonic groups on the microsphere surface exhibit certain surface activity, potentially adsorbing at the oil–water interface and reducing interfacial tension or modifying the interfacial film properties. Furthermore, microsphere adsorption on rock surfaces may alter the pore surface wettability toward a more water-wet state, facilitating oil film detachment and residual oil mobilization. Although this study primarily focuses on the physical plugging and flow diversion effects of microspheres, these potential interfacial contributions warrant further investigation in subsequent research to establish a more comprehensive mechanistic framework for microsphere flooding.

## 4 Conclusions

This study developed AM–AMPS salt-resistant polymer microspheres for high-salinity, low-permeability reservoirs in the B-3 Block of the Ordos Basin, China. Laboratory evaluations of their dynamic and static properties, along with enhanced oil recovery performance, yielded the following key conclusions:

(1) The synthesized microspheres exhibit a uniform spherical morphology and narrow size distribution under high-salinity conditions, with an average particle size of 432.8 nm. Spectral analysis confirms the successful incorporation of AMPS and AM groups, where the strong sulfonic acid groups in AMPS enhance charge density and electrostatic repulsion, thereby imparting excellent structural stability and salt resistance. The microspheres show negligible change in particle size after 90 days of storage, demonstrating long-term stability in high-salinity environments.
(2) The microspheres maintain a highly stable particle size under varying salinity and prolonged conditions, exhibiting excellent salt tolerance and selective plugging capability. In cores with a permeability of 60 × 10^–3^ μm^2^, the system demonstrates good injectivity, with an *RF* of 6.0, a *RRF* exceeding 2.5, and a plugging efficiency of up to 95.86%. By preferentially blocking high-permeability channels (“blocking large pores while sparing small ones”), the microspheres effectively divert fluid flow into low-permeability oil-bearing zones, significantly improving sweep efficiency.
(3) Physical simulation and analysis confirm that microsphere flooding effectively reduces water cut through the synergistic mechanisms of plugging, flow diversion, and profile adjustment. Under high concentration, large injection volume, and low injection rate, the system demonstrates strong adaptability to both heterogeneous and homogeneous reservoirs. It achieves an average oil recovery enhancement of 15–17% in high-salinity, low-permeability reservoirs, offering a practical technical solution and theoretical support for the efficient development of analogous reservoirs.
